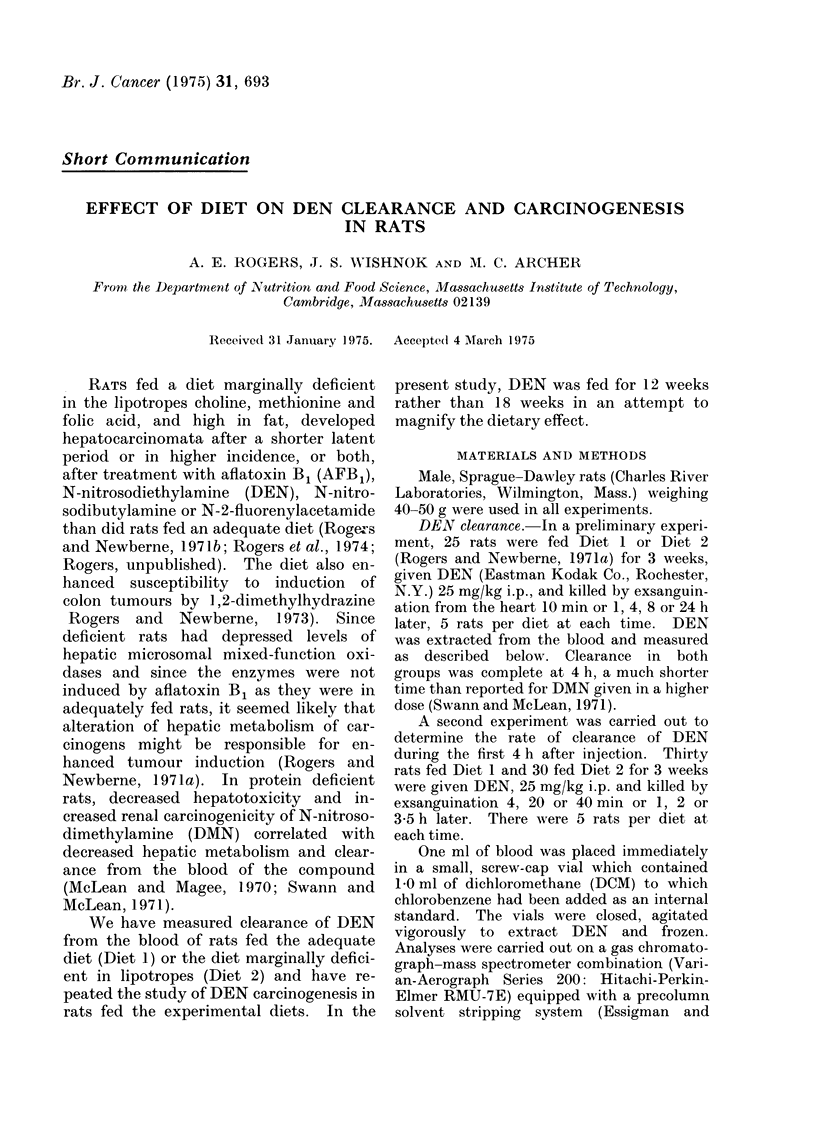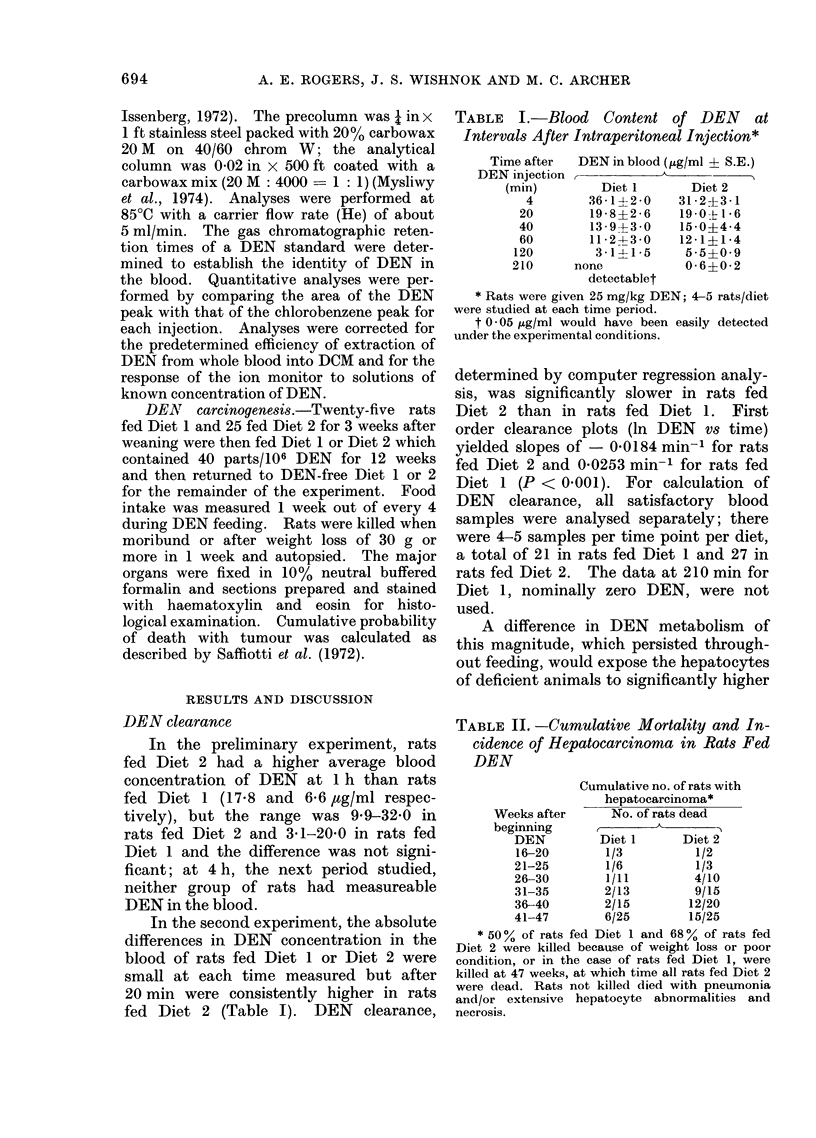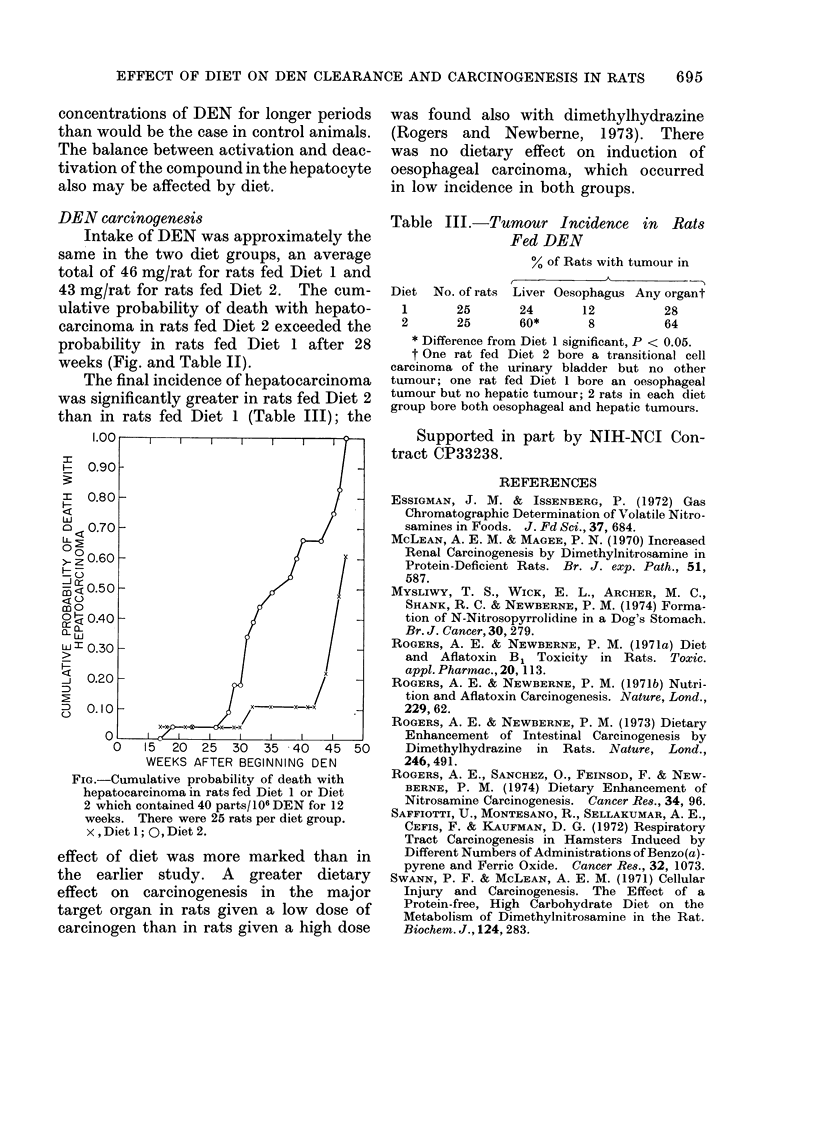# Effect of diet on DEN clearance and carcinogenesis in rats.

**DOI:** 10.1038/bjc.1975.118

**Published:** 1975-06

**Authors:** A. E. Rogers, J. S. Wishnok, M. C. Archer


*Br. J. Cancer* (1975) **31**, 693

***Short Communication***

### EFFECT OF DIET ON DEN CLEARANCE AND CARCINOGENESIS IN RATS

A. E. ROGERS, J. S. WISHNOK AND M. C. ARCHER

*From the Department of Nutrition and Food Science, Massachusetts Institute of Technology, Cambridge, Massachusetts* 02139

Received 31 January 1975. Accepted 4 March 1975

RATS fed a diet marginally deficient in the lipotropes choline, methionine and folic acid, and high in fat, developed hepatocarcinomata after a shorter latent period or in higher incidence, or both, after treatment with aflatoxin $B_1$ ($AFB_1$), N-nitrosodiethylamine (DEN), N-nitrosodibutylamine or N-2-fluorenylacetamide than did rats fed an adequate diet (Rogers and Newberne, 1971*b*; Rogers *et al.*, 1974; Rogers, unpublished). The diet also enhanced susceptibility to induction of colon tumours by 1,2-dimethylhydrazine Rogers and Newberne, 1973). Since deficient rats had depressed levels of hepatic microsomal mixed-function oxidases and since the enzymes were not induced by aflatoxin $B_1$ as they were in adequately fed rats, it seemed likely that alteration of hepatic metabolism of carcinogens might be responsible for enhanced tumour induction (Rogers and Newberne, 1971*a*). In protein deficient rats, decreased hepatotoxicity and increased renal carcinogenicity of N-nitrosodimethylamine (DMN) correlated with decreased hepatic metabolism and clearance from the blood of the compound (McLean and Magee, 1970; Swann and McLean, 1971).

We have measured clearance of DEN from the blood of rats fed the adequate diet (Diet 1) or the diet marginally deficient in lipotropes (Diet 2) and have repeated the study of DEN carcinogenesis in rats fed the experimental diets. In the present study, DEN was fed for 12 weeks rather than 18 weeks in an attempt to magnify the dietary effect.

### MATERIALS AND METHODS

Male, Sprague–Dawley rats (Charles River Laboratories, Wilmington, Mass.) weighing 40–50 g were used in all experiments.

*DEN clearance.*—In a preliminary experiment, 25 rats were fed Diet 1 or Diet 2 (Rogers and Newberne, 1971*a*) for 3 weeks, given DEN (Eastman Kodak Co., Rochester, N.Y.) 25 mg/kg i.p., and killed by exsanguination from the heart 10 min or 1, 4, 8 or 24 h later, 5 rats per diet at each time. DEN was extracted from the blood and measured as described below. Clearance in both groups was complete at 4 h, a much shorter time than reported for DMN given in a higher dose (Swann and McLean, 1971).

A second experiment was carried out to determine the rate of clearance of DEN during the first 4 h after injection. Thirty rats fed Diet 1 and 30 fed Diet 2 for 3 weeks were given DEN, 25 mg/kg i.p. and killed by exsanguination 4, 20 or 40 min or 1, 2 or 3·5 h later. There were 5 rats per diet at each time.

One ml of blood was placed immediately in a small, screw-cap vial which contained 1·0 ml of dichloromethane (DCM) to which chlorobenzene had been added as an internal standard. The vials were closed, agitated vigorously to extract DEN and frozen. Analyses were carried out on a gas chromatograph–mass spectrometer combination (Varian-Aerograph Series 200: Hitachi-Perkin-Elmer RMU-7E) equipped with a precolumn solvent stripping system (Essigman and

Issenberg, 1972). The precolumn was $\frac{1}{4}$ in × 1 ft stainless steel packed with 20% carbowax 20 M on 40/60 chrom W; the analytical column was 0·02 in × 500 ft coated with a carbowax mix (20 M : 4000 = 1 : 1) (Mysliwy *et al.*, 1974). Analyses were performed at 85°C with a carrier flow rate (He) of about 5 ml/min. The gas chromatographic retention times of a DEN standard were determined to establish the identity of DEN in the blood. Quantitative analyses were performed by comparing the area of the DEN peak with that of the chlorobenzene peak for each injection. Analyses were corrected for the predetermined efficiency of extraction of DEN from whole blood into DCM and for the response of the ion monitor to solutions of known concentration of DEN.

*DEN carcinogenesis.*—Twenty-five rats fed Diet 1 and 25 fed Diet 2 for 3 weeks after weaning were then fed Diet 1 or Diet 2 which contained 40 parts/$10^6$ DEN for 12 weeks and then returned to DEN-free Diet 1 or 2 for the remainder of the experiment. Food intake was measured 1 week out of every 4 during DEN feeding. Rats were killed when moribund or after weight loss of 30 g or more in 1 week and autopsied. The major organs were fixed in 10% neutral buffered formalin and sections prepared and stained with haematoxylin and eosin for histological examination. Cumulative probability of death with tumour was calculated as described by Saffiotti *et al.* (1972).

### RESULTS AND DISCUSSION

### *DEN clearance*

In the preliminary experiment, rats fed Diet 2 had a higher average blood concentration of DEN at 1 h than rats fed Diet 1 (17·8 and 6·6 μg/ml respectively), but the range was 9·9–32·0 in rats fed Diet 2 and 3·1–20·0 in rats fed Diet 1 and the difference was not significant; at 4 h, the next period studied, neither group of rats had measureable DEN in the blood.

In the second experiment, the absolute differences in DEN concentration in the blood of rats fed Diet 1 or Diet 2 were small at each time measured but after 20 min were consistently higher in rats fed Diet 2 (Table I). DEN clearance, determined by computer regression analysis, was significantly slower in rats fed Diet 2 than in rats fed Diet 1. First order clearance plots (ln DEN *vs* time) yielded slopes of − 0·0184 $min^{-1}$ for rats fed Diet 2 and 0·0253 $min^{-1}$ for rats fed Diet 1 ($P < 0{\cdot}001$). For calculation of DEN clearance, all satisfactory blood samples were analysed separately; there were 4–5 samples per time point per diet, a total of 21 in rats fed Diet 1 and 27 in rats fed Diet 2. The data at 210 min for Diet 1, nominally zero DEN, were not used.

A difference in DEN metabolism of this magnitude, which persisted throughout feeding, would expose the hepatocytes of deficient animals to significantly higher

TABLE I.—*Blood Content of DEN at Intervals After Intraperitoneal Injection**

| Time after DEN injection (min) | DEN in blood (μg/ml ± S.E.) Diet 1 | Diet 2 |
|---|---|---|
| 4 | 36·1±2·0 | 31·2±3·1 |
| 20 | 19·8±2·6 | 19·0±1·6 |
| 40 | 13·9±3·0 | 15·0±4·4 |
| 60 | 11·2±3·0 | 12·1±1·4 |
| 120 | 3·1±1·5 | 5·5±0·9 |
| 210 | none detectable† | 0·6±0·2 |

* Rats were given 25 mg/kg DEN; 4–5 rats/diet were studied at each time period.

† 0·05 μg/ml would have been easily detected under the experimental conditions.

TABLE II.—*Cumulative Mortality and Incidence of Hepatocarcinoma in Rats Fed DEN*

| Weeks after beginning DEN | Cumulative no. of rats with hepatocarcinoma* / No. of rats dead: Diet 1 | Diet 2 |
|---|---|---|
| 16–20 | 1/3 | 1/2 |
| 21–25 | 1/6 | 1/3 |
| 26–30 | 1/11 | 4/10 |
| 31–35 | 2/13 | 9/15 |
| 36–40 | 2/15 | 12/20 |
| 41–47 | 6/25 | 15/25 |

* 50% of rats fed Diet 1 and 68% of rats fed Diet 2 were killed because of weight loss or poor condition, or in the case of rats fed Diet 1, were killed at 47 weeks, at which time all rats fed Diet 2 were dead. Rats not killed died with pneumonia and/or extensive hepatocyte abnormalities and necrosis.

concentrations of DEN for longer periods than would be the case in control animals. The balance between activation and deactivation of the compound in the hepatocyte also may be affected by diet.

*DEN carcinogenesis*

Intake of DEN was approximately the same in the two diet groups, an average total of 46 mg/rat for rats fed Diet 1 and 43 mg/rat for rats fed Diet 2. The cumulative probability of death with hepatocarcinoma in rats fed Diet 2 exceeded the probability in rats fed Diet 1 after 28 weeks (Fig. and Table II).

The final incidence of hepatocarcinoma was significantly greater in rats fed Diet 2 than in rats fed Diet 1 (Table III); the

FIG.—Cumulative probability of death with hepatocarcinoma in rats fed Diet 1 or Diet 2 which contained 40 parts/$10^6$ DEN for 12 weeks. There were 25 rats per diet group. ×, Diet 1; ○, Diet 2.

effect of diet was more marked than in the earlier study. A greater dietary effect on carcinogenesis in the major target organ in rats given a low dose of carcinogen than in rats given a high dose was found also with dimethylhydrazine (Rogers and Newberne, 1973). There was no dietary effect on induction of oesophageal carcinoma, which occurred in low incidence in both groups.

Table III.—*Tumour Incidence in Rats Fed DEN*

| Diet | No. of rats | % of Rats with tumour in Liver | Oesophagus | Any organ† |
|---|---|---|---|---|
| 1 | 25 | 24 | 12 | 28 |
| 2 | 25 | 60* | 8 | 64 |

* Difference from Diet 1 significant, $P < 0.05$.

† One rat fed Diet 2 bore a transitional cell carcinoma of the urinary bladder but no other tumour; one rat fed Diet 1 bore an oesophageal tumour but no hepatic tumour; 2 rats in each diet group bore both oesophageal and hepatic tumours.

Supported in part by NIH-NCI Contract CP33238.

REFERENCES

ESSIGMAN, J. M. & ISSENBERG, P. (1972) Gas Chromatographic Determination of Volatile Nitrosamines in Foods. *J. Fd Sci.*, **37**, 684.

McLEAN, A. E. M. & MAGEE, P. N. (1970) Increased Renal Carcinogenesis by Dimethylnitrosamine in Protein-Deficient Rats. *Br. J. exp. Path.*, **51**, 587.

MYSLIWY, T. S., WICK, E. L., ARCHER, M. C., SHANK, R. C. & NEWBERNE, P. M. (1974) Formation of N-Nitrosopyrrolidine in a Dog's Stomach. *Br. J. Cancer*, **30**, 279.

ROGERS, A. E. & NEWBERNE, P. M. (1971*a*) Diet and Aflatoxin $B_1$ Toxicity in Rats. *Toxic. appl. Pharmac.*, **20**, 113.

ROGERS, A. E. & NEWBERNE, P. M. (1971*b*) Nutrition and Aflatoxin Carcinogenesis. *Nature, Lond.*, **229**, 62.

ROGERS, A. E. & NEWBERNE, P. M. (1973) Dietary Enhancement of Intestinal Carcinogenesis by Dimethylhydrazine in Rats. *Nature, Lond.*, **246**, 491.

ROGERS, A. E., SANCHEZ, O., FEINSOD, F. & NEWBERNE, P. M. (1974) Dietary Enhancement of Nitrosamine Carcinogenesis. *Cancer Res.*, **34**, 96.

SAFFIOTTI, U., MONTESANO, R., SELLAKUMAR, A. E., CEFIS, F. & KAUFMAN, D. G. (1972) Respiratory Tract Carcinogenesis in Hamsters Induced by Different Numbers of Administrations of Benzo(*a*)-pyrene and Ferric Oxide. *Cancer Res.*, **32**, 1073.

SWANN, P. F. & McLEAN, A. E. M. (1971) Cellular Injury and Carcinogenesis. The Effect of a Protein-free, High Carbohydrate Diet on the Metabolism of Dimethylnitrosamine in the Rat. *Biochem. J.*, **124**, 283.